# Modulation of dendritic cell alternative activation and function by the vitamin A metabolite retinoic acid

**DOI:** 10.1093/intimm/dxv020

**Published:** 2015-04-20

**Authors:** Lucy H. Jones, Peter C. Cook, Alasdair C. Ivens, Graham, D. Thomas, Alexander T. Phythian-Adams, Judith E. Allen, Andrew S. MacDonald

**Affiliations:** ^1^Institute of Immunology and Infection Research, Centre for Immunity, Infection and Evolution, School of Biological Sciences, University of Edinburgh, Scotland, UK; ^2^Manchester Collaborative Centre for Inflammation Research, Faculty of Life Sciences, University of Manchester, Manchester M13 9NT, UK

**Keywords:** APC, IL-4, T_h_2

## Abstract

The archetypal T_h_2 cytokine IL-4 has previously been shown to alternatively activate murine macrophages and, more recently, dendritic cells (DCs) both *in vitro* and *in vivo.* IL-4 has also been shown to induce *Aldh1a2* (aldehyde dehydrogenase 1a2) expression in murine macrophages recruited to the peritoneal cavity. However, the influence of IL-4 on DC *Aldh1a2* induction *in vivo* has not yet been addressed. In this work, we found that DCs show enhanced aldehyde dehydrogenase enzyme activity *in vivo*, which led us to investigate the impact of the vitamin A metabolite all-*trans* retinoic acid (RA) on DC alternative activation and function. Antagonism of RA receptors reduced production of resistin-like molecule alpha by DCs responding to IL-4, while addition of exogenous RA enhanced production of this marker of alternative activation. Functionally, RA increased DC induction of CD4^+^ T-cell IL-10, while reducing CD4^+^ T-cell IL-4 and IL-13, revealing a previously unidentified role for RA in regulating the ability of alternatively activated DCs to influence T_h_2 polarization.

## Abstract (precis)

Retinoic acid modulates the functions of IL-4 in alternatively activated DCs

## Introduction

The importance of vitamin A (Retinol) signalling within the immune system has been established during the past decade and highlighted recently by work indicating that retinoic acid (RA), the transcriptionally active metabolite of vitamin A, is involved in the development and promotion of CD4^+^ T-cell responses (1–3). RA expression by gut-associated dendritic cells (DCs) imprints intestinal specificity on T cells (2, 4, 5), and mucosal CD103^+^ DCs have been shown to express *Aldh1a2* (aldehyde dehydrogenase 1a2; RALDH), a Retinol metabolizing enzyme (3), and promote extra-thymic conversion of naive T cells into Foxp3^+^ regulatory T cells (3, 6). Additionally, it is becoming clear that vitamin A signalling is required for T helper cell function beyond the promotion of FoxP3 expression (7–10).

IL-4 has been shown to alter DC cytokine production and modulate their capacity to polarize naive T cells (11–15). Further, IL-4 responsiveness is known to be required for optimal DC polarization of CD4^+^ T cells, as IL-4Rα-deficient DCs are less competent inducers of T-cell IL-10, IL-17 and IFNγ (15). In addition, DCs undergo a programme of alternative activation in response to IL-4, characterized by high-level production of the resistin-like molecule alpha (RELMα) (15, 16). Expression of RELMα by alternatively activated DCs (AADCs) during T_h_2 priming regulates IFNγ and IL-4 and promotes IL-10 and IL-13 secretion by CD4^+^ T cells (15). IL-4 can also induce DC *Aldh1a2* expression/aldehyde dehydrogenase activity (17–19), and it has recently been suggested that there is interplay between RA and IL-4 in DC expression of *Arg1* (20), *Aldh1a2* and induction of Foxp3^+^ Tregs in the context of experimental autoimmune encephalomyelitis (19). However, crosstalk between RA and IL-4 in the process of DC alternative activation, and how this may affect T_h_2 polarization, has not yet been addressed.

In this study, we have investigated the combined impact of RA and IL-4 on DC alternative activation and function. We first assessed the influence of IL-4 on the mRNA profile of murine bone marrow-derived DCs (BMDCs) *in vitro* and found that this resulted in significantly altered expression of 109 genes, with *Aldh1a2* being among 21 genes up-regulated >2-fold following exposure to IL-4. Aldehyde dehydrogenase activity was also induced in DCs, but not macrophages, following *in vivo* delivery of IL-4. Importantly, we reveal that RA promotes DC RELMα production and regulates the ability of AADCs to support T_h_2 cell polarization. Taken together, our data provide compelling evidence that concurrent exposure to IL-4 and RA results in phenotypic and functional changes in DCs, in particular modifying their capacity to polarize CD4^+^ T cells.

## Methods

### Mice

C57BL/6, BALB/c, IL-10eGFP (21) and 4get (22) (IL-4GFP; BALB/c) mice were maintained at the University of Edinburgh. All experiments were approved under a Project License granted by the Home Office (UK) and conducted in accordance with local guidelines.

### *In vivo* IL-4c

C57BL/6 female mice were injected intra-peritoneally with PBS or IL-4 complex (IL-4c) that is composed of rIL-4 (Peprotech, UK) complexed at a 1:5 molar ratio with anti-IL-4 antibody (Clone 11B11, BioXcell, Malaysia). Mice received 0.1, 0.5 or 1 μg of IL-4 and cells were harvested 6h later.

### DC culture

BMDCs were generated with 20-ng ml^−1^ recombinant granulocyte macrophage colony-stimulating factor (rGM-CSF) as previously described (23), with the omission of 2-mercaptoethanol. Following 10 days of culture, immature cells were cultured for a final 6 or 18h with or without rIL-4 (20ng ml^−1^; Peprotech), all-*trans* RA reconstituted in DMSO (RA; 10 μM, Sigma, UK) or LE540 (10 μM, WAKO, Japan) reconstituted in DMSO, and cRPMI-1640 containing 5-ng ml^−1^ rGM-CSF (Peprotech).

### RNA extraction and Illumina BeadChip arrays

RNA was extracted from cells using TRIzol or Pure Link RNA Mini Kit and DNase-treated according to the manufacturer’s instructions (Life Technologies, UK). RNA was converted to cDNA using Superscript-III (Invitrogen, UK). Quantitative PCR was performed using SYBR Green mastermix-II (Roche, UK) and a Roche Light Cycler 480, using primers previously documented (15) with the exception of *Aldh1a2*F:5′-CATGGTATCCTCCGCAATG-3′ and *Aldh1a2*R:5′GCGCATTTAAGGCATTGTAAC-3′. To assess DC gene expression, RNA was labelled using the TotalPrep RNA Amplification kit (Life Technologies) and hybridized to Illumina MouseWG-6 BeadChip arrays (MouseWG6_V2_0_R3_11278593_A, each comprising 45281 features), according to the manufacturer’s instructions. Three biological replicates were carried out for wild-type (WT) DCs. All analyses were conducted in R using Bioconductor packages (www.r-project.org; www.bioconductor.org). A total of six arrays were quality controlled (QC) analysed using the arrayQualityMetrics package in Bioconductor (24, 25). Raw data from arrays that passed QC were transformed using a variance-stabilizing transformation method prior to normalization across all arrays using the robust spline normalization method, using the lumi package in Bioconductor. Pairwise group comparisons were undertaken using linear modelling. Subsequently, empirical Bayesian analysis was applied, including vertical (within a given comparison) *P* value adjustment for multiple testing, which controls for false discovery rate, using the limma Bioconductor package (25). The data discussed in this publication have been deposited in NCBI’s Gene Expression Omnibus (26) and are accessible through GEO Series accession number GSE59868 (http://www.ncbi.nlm.nih.gov/geo/query/acc.cgi?acc=GSE59868).

### Aldefluor assay

For select experiments, the aldefluor assay (Stemcell Technologies) was performed, following surface staining, essentially as in manufacturer’s instructions.

### Flow cytometry

BMDCs described in Fig. 2 were flow sorted based on CD11c and aldefluor activity using a BD FACS Aria-II. Spleen and lymph node suspensions from 4Get [IL-4eGFP (22)] or TIGR [IL-10eGFP (21)] mice were sorted as green fluorescent protein (GFP)^−^CD4^+^ using a BD FACS Aria-II. peritoneal exudate cells were stained directly *ex vivo*, essentially as in (15). Samples were acquired using an LSR II or FACS Canto II flow cytometer, using BD FACS Diva software and analysed with FlowJo v.9 software (Tree Star, Inc., USA).

### RELMα and Ym1 intracellular staining

Cells were surface stained and washed prior to fixation using 2% formaldehyde, permeabilized on ice for 20min using 1× Permeabilization buffer (BD Bioscience) prior to primary antibody staining using polyclonal rabbit antibodies against murine RELMα (Peprotech) and biotinylated polyclonal goat IgG against murine Ym1/ECF-L (R&D Systems). The Zenon Rabbit IgG labelling kit (Life Technologies) was used to detect bound anti-RELMα, and Streptavidin-allophycocyanin was used to detect bound anti-Ym1Biotin (eBioscience) (15).

### DC:T-cell co-culture

Fifty thousand sorted IL10-eGFP^−^CD4^+^ or 4get^−^CD4^+^ were cultured with 2500 BMDCs, with 1-μg ml^−1^ anti-CD3 (clone 145-2C11 produced in house) with or without IL-4 (20ng ml^−1^; Peprotech) and RA (10 μM; Sigma) in cRPMI-1640. Cells were cultured at 37°C in a humidified atmosphere of 5% CO_2_ for 4 days.

### ELISA

Cytokine ELISAs were performed on culture supernatants using paired mAb purified in house, or purchased from eBioscience, BD Pharmingen or R&D Systems (UK), and recombinant cytokine standards purchased from Peprotech. Ym1 ELISAs were performed using a DuoSet ELISA kit (R&D Systems), RELMα ELISAs were performed using paired rabbit anti-murine RELMα antibodies (Peprotech) and standards were made using recombinant murine RELMα (Peprotech).

### Statistical analyses

Statistical analyses were carried out using GraphPad Prism 5 software one-way analysis of variance with Dunnett’s post-test or Student’s *t*-tests were employed to determine significant differences between groups: *P < 0.05, **P < 0.01, ***P < 0.001, ****P < 0.0001.

## Results and Discussion

### IL-4 enhances DC aldehyde dehydrogenase activity and RELMα protein expression *in vitro* and *in vivo*

WT DCs were cultured *in vitro* with rIL-4, RNA extracted and an Illumina mRNA expression array carried out (Supplementary Figure 1, available at *International Immunology* Online). Upon KEGG enrichment, the pathway with which the IL-4-induced gene profile most closely associated was ‘metabolic pathways’, in which 14 genes were significantly increased (Supplementary Table 1, available at *International Immunology* Online). In order to determine genes that may have more prominent roles in modulating cellular function following exposure to IL-4, we chose to use a 2-fold change (2FC) cut-off, focusing only on significantly altered transcripts (adj*P* = <0.05) that doubled in expression in response to IL-4 (Supplementary Figure 1B, available at *International Immunology* Online). When using this level of stringency, only two metabolic genes remained: *Ak2* (adenylate kinase 2), a mitochondrial protein (27), and *Aldh1a2* (Supplementary Figure 1C, available at *International Immunology* Online). In keeping with our previous identification of the ability of IL-4 to alternatively activate DCs (15), the genes for *Ccl24*, *Chi3l3*, *Chi3l4* and *Retnla* were all significantly up-regulated >2FC within the 6-h IL-4-treated BMDC microarray (Supplementary Figure 1C, available at *International Immunology* Online), whereas *Arg1* was not significantly altered (adj*P* = <0.05) at any fold cut-off.

**Fig. 1.**
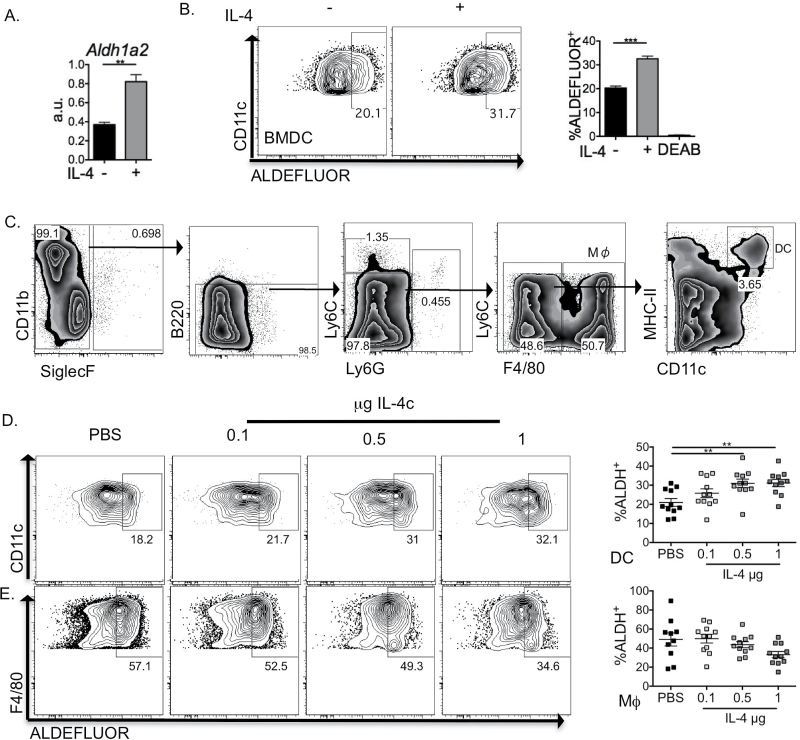
IL-4 induces aldehyde dehydrogenase activity and alternative activation in DCs *in vitro* and *in vivo*. BMDCs were cultured overnight with 20-ng ml^−1^ IL-4 (A and B) and expression of *Aldh1a2* (A) and aldehyde dehydrogenase activity (B) assessed. IL-4c (doses as described in figure) was injected intra-peritoneally. Peritoneal exudate cells were harvested 6h later, DCs were defined as SiglecF^−^B220^−^Ly6C^−^Ly6G^−^F4/80^−^CD11c^+^MHCII^hi^ and macrophages as SiglecF^−^B220^−^Ly6C^−^Ly6G^−^F4/80^+^ (C). Aldehyde dehydrogenase activity of DC and macrophage populations was assessed *ex vivo* (D). Data in A and B are representative of five independent experiments, error bars represent triplicate culture wells. Data points in (D) and (E) represent individual mice, pooled from three independent experiments *n* = 10–11 per group. **P < 0.01, ***P < 0.001.

Confirming our mRNA expression array data (Supplementary Figure 1, available at *International Immunology* Online), *in vitro* culture of DCs with rIL-4 resulted in significant enhancement of *Aldh1a2* transcript, as measured by quantitative PCR (Fig. 1A). Furthermore, enhanced *Aldh1a2* transcription following IL-4 exposure correlated with increased DC aldehyde dehydrogenase activity (28) (Fig. 1B), as has been suggested previously (17–19).

**Fig. 2.**
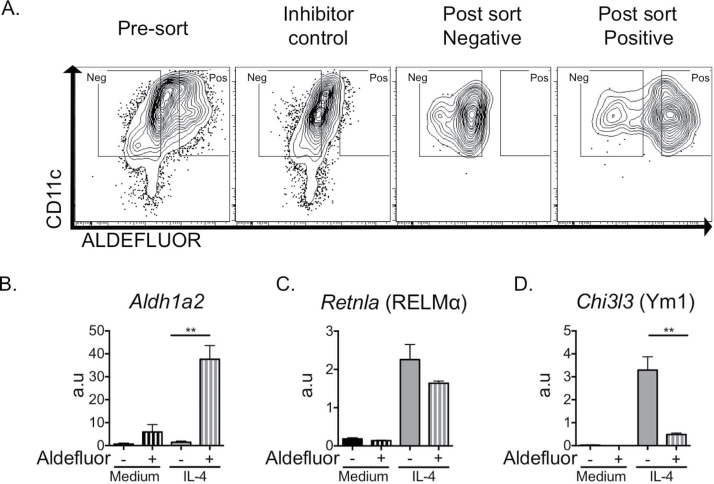
IL-4-induced DC aldehyde dehydrogenase activity correlates with expression of RELMα but not Ym1. Following overnight culture with IL-4, BMDC populations were sorted by flow cytometry based upon aldefluor activity (A) and quantitative PCR was used to assess expression of *Aldh1a2* (B), *Retnla* (C) and *Chi3l3* (D). Data are representative of 4–5 independent experiments. Error bars represent triplicate culture wells, a.u. = arbitrary units as compared with *Hprt* expression. **P < 0.01.

Next, to determine the impact of IL-4 on DC retinoid metabolism *in vivo*, we injected IL-4c (29) into the peritoneal cavity of C57BL/6 mice and assessed DC aldehyde dehydrogenase activity (Fig. 1C). Peritoneal DCs, defined as SiglecF^−^B220^−^ Ly6C^−^Ly6G^−^F4/80^−^CD11c^+^MHCII^hi^ (Fig. 1C), displayed aldehyde dehydrogenase activity (Fig. 1D) in an IL-4c dose-dependent manner. This was in contrast to resident macrophages of the peritoneal cavity (Fig. 1D), which failed to increase aldehyde dehydrogenase activity in response to IL-4c treatment (30). We then used flow cytometric cell sorting based on aldefluor activity (Fig. 2A) to confirm that IL-4-exposed DCs possessing aldehyde dehydrogenase activity expressed high levels of *Aldh1a2* (Fig. 2B). Furthermore, IL-4-exposed aldefluor positive DCs also expressed high levels of *Retnla* (Fig. 2C). However, in contrast to *Retnla*, aldehyde dehydrogenase activity inversely correlated with *Chi3l3* expression (Fig. 2D). These results reveal IL-4-dependent induction of aldehyde dehydrogenase activity in DCs *in vivo* (Fig. 1C), confirming our *in vitro* data (Fig. 1) and previous *in vitro* reports (17–19, 31). Further, these results also show that IL-4 can induce aldehyde dehydrogenase activity and *Retnla* expression within the same DC population (Fig. 2).

Enhanced expression of RALDH2, MHC-II and CD206 in myeloid cells of monocytic but not tissue resident origin has recently been shown (30). Our new data suggest that within a population of DCs, a spectrum of expression of such markers can occur, as DCs sorted as being aldefluor positive showed reduced expression of *Chi3l3* (and *Mrc1*, data not shown) compared with their aldefluor negative counterparts (Fig. 2). Furthermore, understanding the interaction between RA and IL-4 in cells of myeloid origin is extremely timely given the recent report that RA controls the tissue-specific localization of peritoneal macrophages (32), and our data that IL-4 drives a program of proliferation within macrophages of the same tissue site (33).

### Inhibition of retinoic acid receptors reduces IL-4-dependent RELMα production by DCs

**Fig. 3.**
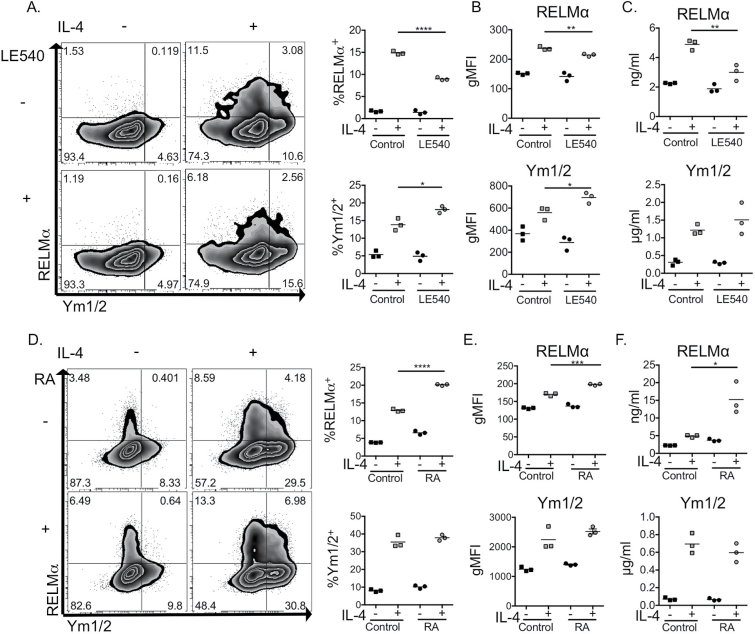
RAR signalling regulates IL-4-driven DC alternative activation. BMDCs were cultured overnight with or without 20-ng ml^−1^ IL-4 and either the pan RAR antagonist LE540 (A–C), RA (D–F) or vehicle control. RELMα and Ym1/2 protein production was assessed by intracellular staining (A and D), while RELMα and Ym1/2 secretion was assessed by ELISA (C and F). Data are representative of 3 (A–C) and >5 (D–F) independent experiments. Error bars represent triplicate culture wells. *P < 0.05, **P < 0.01, ****P < 0.0001.

To begin to understand the interaction between alternative activation and retinoid metabolism in DCs, we used the pan retinoic acid receptor (RAR) antagonist LE540 (34) to inhibit RAR function. DCs were cultured simultaneously with LE540 and IL-4 and their alternative activation status assessed by intracellular staining (Fig. 3A and B) and ELISA (Fig. 3C). In comparison with cells that were not treated with the RAR antagonist, LE540 limited IL-4 induction of intracellular RELMα protein (Fig. 3A and B) and reduced RELMα secretion by DCs (Fig. 3C). Again, in contrast to RELMα, LE540 did not impair intracellular or secreted Ym1/2 in DCs treated with IL-4 (Fig. 3A–C).

Having identified that RA signalling enhanced DC RELMα production, we next wanted to directly address whether RA was able to promote RELMα production in the context of IL-4. To test this, we exposed DCs to exogenous RA either alone or in the presence of IL-4 and assessed levels of intracellular (Fig. 3D and E) and secreted (Fig. 3F) RELMα and Ym1/2. The addition of RA alone had no significant impact on DC expression of the alternative activation markers RELMα or Ym1/2 (Fig. 3D–F). However, culture with both RA and IL-4 resulted in a significantly higher proportion of DCs that synthesized (Fig. 3D and E) and secreted (Fig. 3F) RELMα protein than those exposed to IL-4 alone. In contrast, exogenous RA did not significantly alter DC production of Ym1/2 (Fig. 3D–F). Taken together, these data indicate that retinoid availability influences the alternative activation profile of DCs. Exogenous RA, likely acting via RARs, was able to enhance IL-4-induced RELMα, whereas exogenous RA had no significant impact on DC Ym1 capacity (Fig. 3). Thus, our data support a central role for RA and RAR signalling in RELMα, but not Ym1/2, production by AADCs.

RA signals via binding to heterodimeric nuclear hormone receptors of the RAR or retinoic X receptor (RXR) families (35). Ligand-bound RAR/RXRs associate with RA response elements in the promoter regions of target genes to mediate their downstream effects (36). Although RARα is the dominant RAR isoform in myeloid cells (37), all RAR isoforms have been detected in murine splenic DCs (38) and RARβ has recently been shown to interact with the *Il4ra* promoter (19). One hypothesis to explain the ability of exogenous RA or inhibition of RAR signalling to differentially influence RELMα and Ym1/2 (Fig. 3) is that different nuclear pairings of RARs (α, β, γ)/RXRs may be responsible for controlling distinct aspects of the profile of alternative activation that is initiated by IL-4 in DCs.

Up-regulation of RELMα but not Ym1/2 in the presence of RA, a molecule known to be involved in induction of immune regulation/tolerance (1), is consistent with the putative functions of these two molecules. Ym1 is thought to be pro-inflammatory, forming epithelial damage-associated crystals in the lung (39), causing neutrophilic inflammation during nematode infection (40) and increasing in response to stab wounding or chemical-induced seizure in the brain (41). In contrast, RELMα can be described as a regulatory molecule, having been shown to limit pulmonary inflammation (42) and promote IL-10 secretion (15). RA may thus be able to fine-tune AADC function at sites of inflammation by enhancing IL-4-driven regulatory, but not inflammatory, molecules.

### RA and IL-4 alter T-cell polarization by DCs

**Fig. 4.**
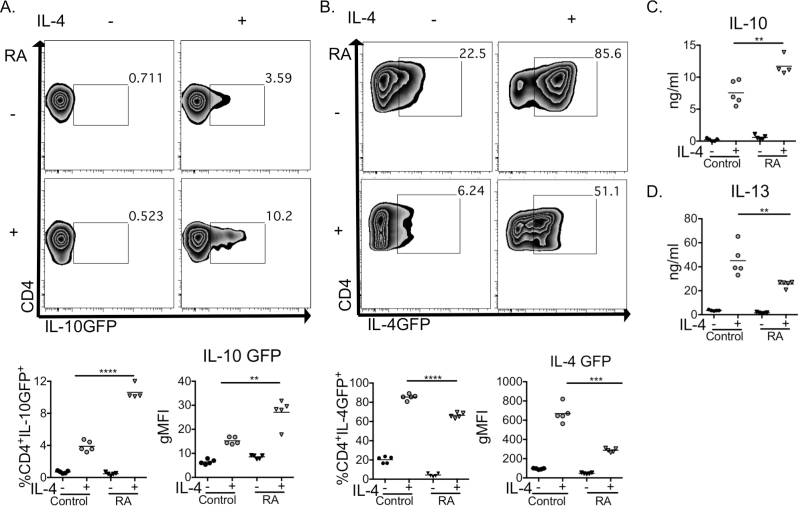
RA and IL-4 alter DC APC capacity. IL-10eGFP^−^CD4^+^ or 4Get^−^CD4^+^ T cells were cultured for 4 days with WT granulocyte macrophage colony stimulating factor derived BMDCs, anti-CD3 mAb, with or without 20-ng ml^−1^ IL-4, and RA and assessed for IL-10 (A) or IL-4 (B) mRNA expression by flow cytometry and IL-10 (C) and IL-13 (D) secretion by ELISA. Data are representative of two (A and B) or four (C and D) independent experiments, *n* = 4–5 replicate wells per condition. **P < 0.01, ***P < 0.001, ****P < 0.0001.

Previous studies have suggested that RA can directly influence CD4^+^ T-cell polarization, by promoting IL-4 secretion under T_h_2 conditions (43, 44) or limiting IFNγ from already polarized T_h_1 cells (45). However, these early reports did not assess the influence of RA on T cells in the presence of DCs. As RA and IL-4 enhanced DC RELMα production (Fig. 3), and we have previously shown that *Retnla* ^−/−^ DCs display impaired ability to induce CD4^+^ T-cell IL-10 and IL-13 secretion (15), we investigated the induction of T_h_2 cytokines by DCs exposed simultaneously to RA and IL-4 (Fig. 4). DC:T-cell co-culture assays incorporating 4Get [IL-4eGFP (22)] or TIGR [IL-10eGFP (21)] reporter T cells and WT DCs allowed us to determine if DCs exposed to RA and IL-4 had an altered capacity to polarize polyclonally activated T cells (Fig. 4). In agreement with our previous work (15), IL-4 enhanced DC induction of CD4^+^ T-cell IL-10, as assessed by GFP expression and IL-10 protein secretion (Fig. 4). Notably, while IL-10 levels increased further upon simultaneous addition of RA (Fig. 4A–C), the addition of RA reduced *Il4* mRNA transcription in CD4^+^ T cells, as assessed by GFP expression (Fig. 4B). Due to the exogenous recombinant IL-4 in this system, it was not possible to accurately measure T-cell IL-4 secretion. However, RA exerted a similar regulatory effect on the ability of DCs to support T-cell IL-13 production, with significantly reduced IL-13 secretion following addition of RA along with IL-4, as compared with IL-4 alone (Fig. 4D). These data strongly suggest that, in the context of RA, AADCs have a modified ability to promote T_h_2 effector responses, with RA altering the profile of responding CD4^+^ T cells such that they produce lower levels of IL-4 and IL-13, and increased IL-10.

Although we cannot exclude the possibility that part of the impact of RA in these experiments may be through its direct action on responding T cells, our novel data indicating that vitamin A metabolites alter DC ability to promote or inhibit several key facets of T_h_2 cytokine production (Fig. 4) are supported by a recent report where DCs isolated from vitamin A-deficient animals were shown to induce higher levels of CD4^+^ T-cell IL-13 in a partially IL-6-dependent manner (10). Our data are also consistent with a recent study showing that the immune system can act as a sensor of malnutrition, promoting the secretion of IL-13 from ILC2s in the absence of sufficient RA (46). Our work reveals that DCs exposed to both RA and IL-4 express increased levels of RELMα, as compared with IL-4 alone (Fig. 3). Since we have previously shown that RELMα regulates CD4^+^ T-cell responses via the promotion of IL-13 and IL-10 and the inhibition of IL-4 (15), we anticipated that we would see an increase in both IL-10 and IL-13, and reduced IL-4 secretion, by T cells stimulated in the presence of DCs, RA and IL-4. In keeping with this hypothesis, T-cell transcription of IL-4 decreased and production of IL-10 increased following co-culture with RA-treated AADCs (Fig. 4A–C). However, contrary to expectation, IL-13 was found to decrease in the presence of both RA and IL-4 (Fig. 4D), revealing an additional role for RA in IL-13 inhibition. In our hands, IL-4 and RA down-regulated DC secretion of IL-6 (data not shown), which may contribute to reduced T-cell IL-13 production upon co-culture, as blocking IL-6 in CD8α^−^CD11b^+^CD103^−^ MLN DC:T-cell cultures has been shown to limit IL-13 secretion (10). These data raise the interesting possibility that the balance of alternative activation products such as RELMα, with vitamin A metabolites such as RA, can significantly influence the functional capacity of DCs to polarize T cells.

This work elevates current understanding of the impact of environmental mediators on DC activation and function, in particular highlighting the singular and collective influence of RA and IL-4 over DC alternative activation and ability to direct T_h_2 responses. These findings may be particularly relevant when considering that countries with high levels of dietary vitamin A deficiency are also regions in which type-2 infections are prevalent (47).

## Supplementary data

Supplementary data are available at *International Immunology* Online.

## Funding

Wellcome Trust studentships to L.H.J. and G.D.T.; MRC programme grant to J.E.A.; MRC Senior Non-Clinical Fellowship to A.S.M. (G0701437).

## Supplementary Material

Supplementary Data
