# Supplementary material for: Modulation of dendritic cell alternative activation and function by the vitamin A metabolite retinoic acid
Source: Int Immunol. 2015 Apr 20;27(11):589–96. doi: 10.1093/intimm/dxv020 (PMC4625886; doi:10.1093/intimm/dxv020)
Supplement: Supplementary Data [file supp_27_11_589__index.html]

Modulation of dendritic cell alternative activation and function by the vitamin A metabolite retinoic acid — Modulation of dendritic cell alternative activation and function by the vitamin A metabolite retinoic acid — Modulation of dendritic cell alternative activation and function by the vitamin A metabolite retinoic acid — Supplementary Data 

# Modulation of dendritic cell alternative activation and function by the vitamin A metabolite retinoic acid

## Supplementary Data

Data files

**Files in this Data Supplement:**

- Supplementary Data - Supplementary Data
- Supplementary Data - Supplementary Data
