# Supplementary figures and images for: Modulation of dendritic cell alternative activation and function by the vitamin A metabolite retinoic acid
Source: Int Immunol. 2015 Apr 20;27(11):589–96. doi: 10.1093/intimm/dxv020 (PMC4625886; doi:10.1093/intimm/dxv020)

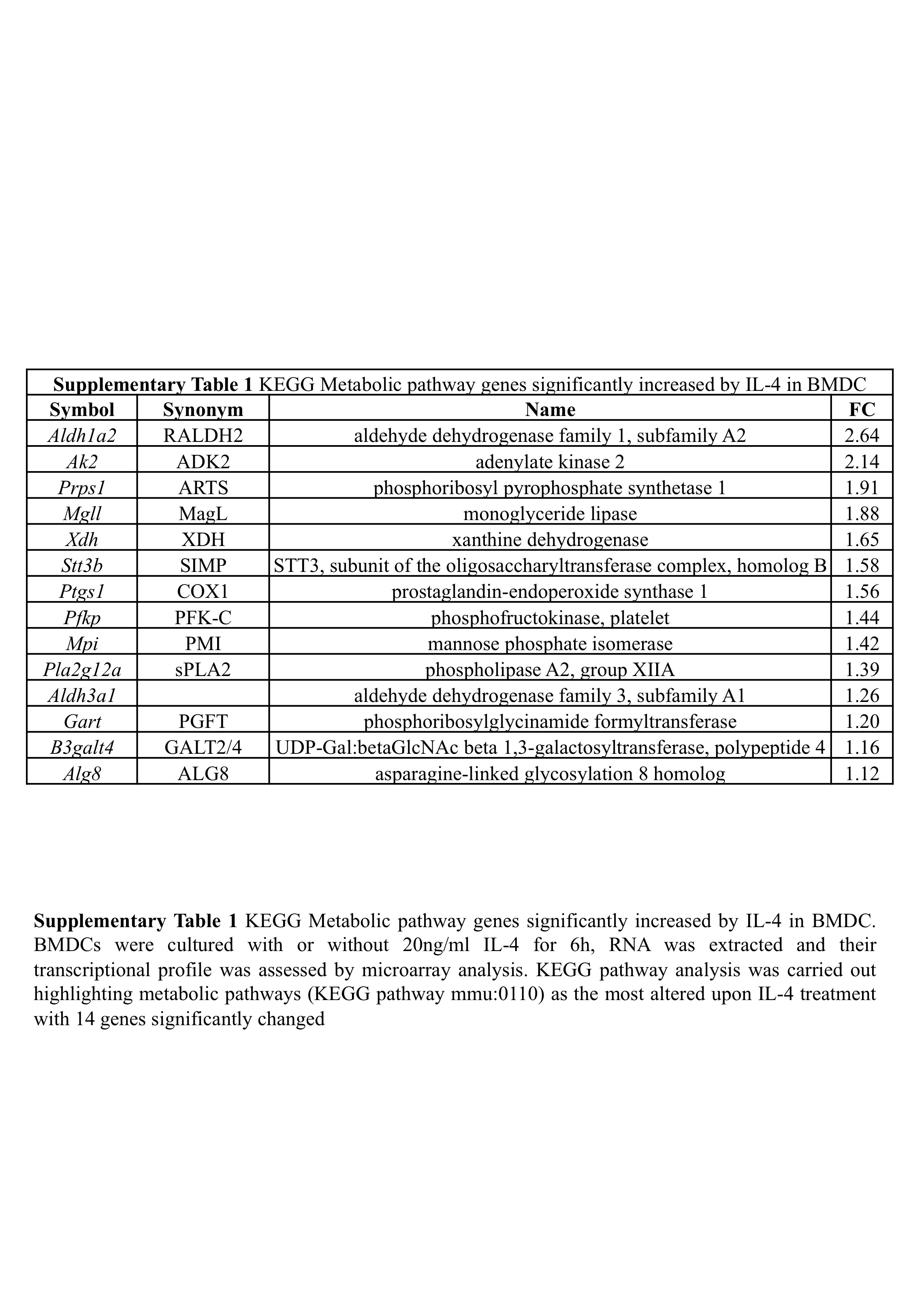

Supplement: Supplementary Data [file supp_dxv020_Supp_Table_1.jpg]

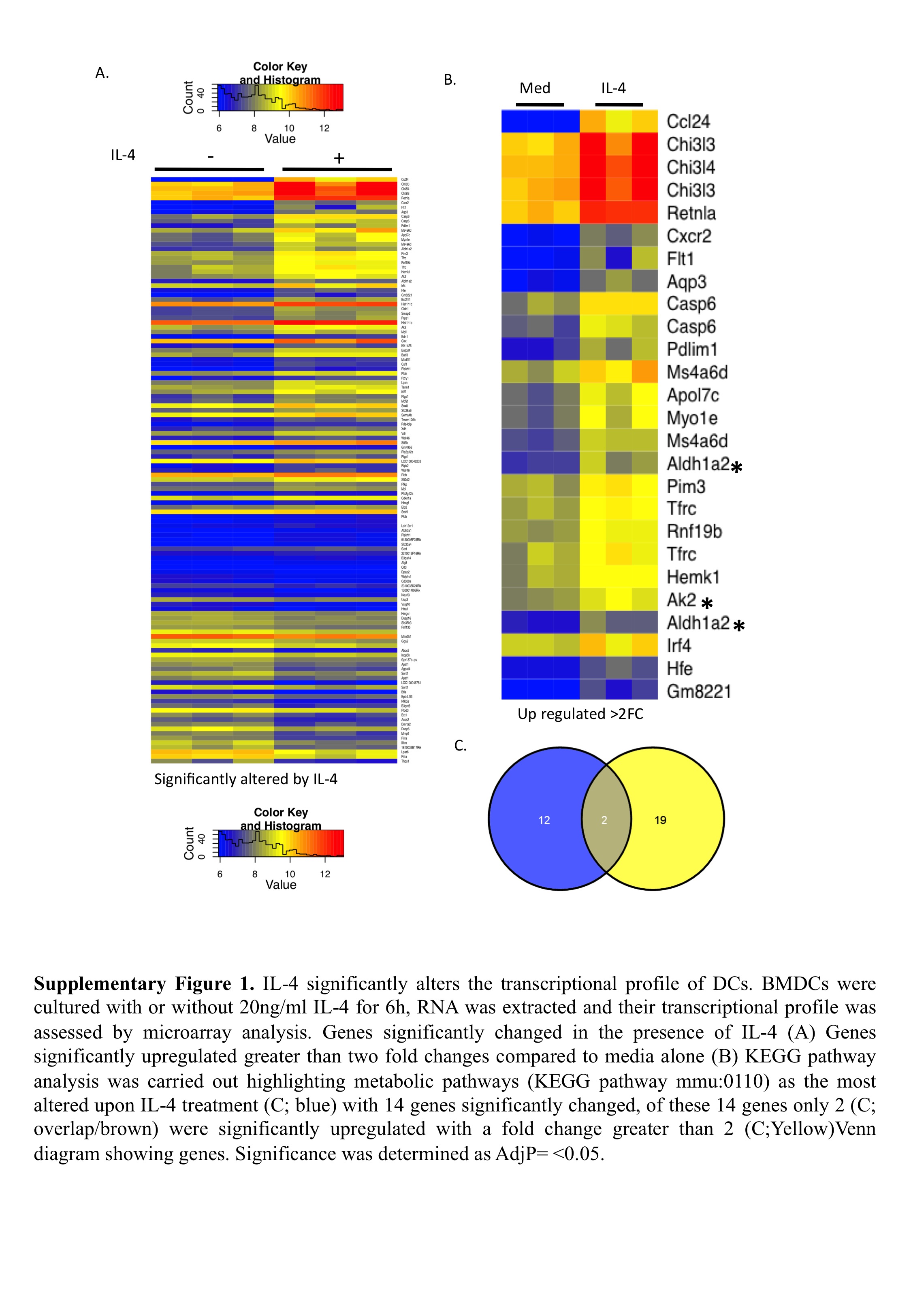

Supplement: Supplementary Data [file supp_dxv020_SuppFig1.jpg]
